# Polymorphisms in the promoter region of the *CRBN* gene as a predictive factor for the first-line CTD therapy in multiple myeloma patients

**DOI:** 10.18632/oncotarget.25307

**Published:** 2018-05-08

**Authors:** Aneta Szudy-Szczyrek, Radosław Mlak, Michał Szczyrek, Sylwia Chocholska, Jacek Sompor, Adam Nogalski, Teresa Małecka-Massalska, Marek Hus

**Affiliations:** ^1^ Department of Haematooncology and Bone Marrow Transplantation, Medical University of Lublin, 20-081 Lublin, Poland; ^2^ Department of Human Physiology, Medical University of Lublin, 20-080 Lublin, Poland; ^3^ Department of Internal Medicine in Nursing, Medical University of Lublin, 20-090 Lublin, Poland; ^4^ Department of Pneumology, Oncology and Allergology, Medical University of Lublin, 20-950 Lublin, Poland; ^5^ Department of Trauma Surgery and Emergency Medicine, Medical University of Lublin, 20-081 Lublin, Poland

**Keywords:** multiple myeloma, thalidomide, cereblon, polymorphism

## Abstract

Cereblon is a primary molecular target for immunomodulatory drugs. The aim of this study was to evaluate the influence of selected clinical and molecular factors including single nucleotide polymorphisms (SNPs) in *CRBN* gene on the efficacy of first line CTD (cyclophosphamide, thalidomide, dexamethasone) chemotherapy in patients with multiple myeloma. Study group consisted of 68 patients. Analysis of CRBN gene SNPs (rs6768972, rs1672753) was performed using Real-Time PCR genotyping technique. Median progression free survival (PFS) was 15 months and overall survival (OS) 79 months. Factors associated with significantly shorter OS included ISS 3, kidney disease, weight loss, anemia, thrombocytopenia, hypoalbuminemia, elevated β2-microglobuline and CRP. The presence of t(4;14) was associated with significantly shorter PFS and OS. Both examined SNPs proved to be statistically significant, independent predictive factors of efficacy of the CTD chemotherapy. The presence of AA genotype (rs6768972) correlated with longer median PFS (18 vs 9 months; HR=0.49,95% CI: 0.26-0.91, p=0.0062). Conversely, in the carriers of CC genotype (rs1672753) significantly shorter median PFS was observed (4 vs 16 months; HR=3.93, 95% CI: 0.26-59.64, p=0.0321). In conclusion, SNPs of the *CRBN* gene may be useful in qualifying patients for treatment with regimens containing thalidomide.

## INTRODUCTION

Multiple myeloma (MM) is a malignant disease characterized by clonal proliferation of atypical plasma cells, which is usually accompanied by the expression of an abnormal monoclonal protein. It constitutes about 0.8% of all malignant neoplasms and is the third most prevalent cancer of the hematopoietic system. The progress in the treatment of MM that has occurred during the last 30 years is attributed to the introduction of immunomodulatory drugs (IMiDs) and proteasome inhibitors. According to the most recent data, 5-year survival in MM patients reached 48.5% and median OS exceeded 6 years. However, the disease remains incurable [1, 2].

Thalidomide, a drug with multidirectional activity towards bone marrow microenvironment and against myeloma cells was the first of IMiDs used in the therapy of MM. It directly affects the neoplastic plasma cells, stopping the cells in G1 phase and causing apoptosis. Furthermore, it inhibits the processes of angiogenesis and adhesion of myeloma cells to the bone marrow stroma. As a result of blocking the production and release of cytokines and growth factors, it inhibits the plasma cells development and migration. Moreover, it exhibits immunomodulatory activity associated with the induction of helper Th1 lymphocytes, excretion of interferon γ (IFN−γ) and interleukin 2 (IL−2) [3, 4].

The key to understanding thalidomide’s mechanism of action came with the discovery of cereblon (CRBN), which constitutes the common molecular target for IMiDs and is responsible both for their antiproliferative, antiangiogenic and immunomodulatory activity, and the occurrence of teratogenic adverse effects [5, 6]. It has been proven that high CRBN expression in patients with MM is associated with good clinical response to IMiDs, while inactivation of *CRBN* gene in cell lines and low protein expression determines resistance to treatment [7, 8].

The aim of the study was to identify potential factors that might affect individual response to chemotherapy based on thalidomide in combination with cyclophosphamide and dexamethasone (CTD). The study analyzed selected clinical and molecular factors including single nucleotide polymorphisms (SNPs) of the *CRBN* gene. Effect of SNPs of genes encoding proteins involved in drug metabolism is currently a very attractive subject of research in cancer biology. It has been shown that the genetically conditioned response to drugs, depending among others on SNPs, is one of the most important factors influencing the efficacy and safety of anticancer therapy [9, 10, 11]. Two investigated polymorphic sites: c.-414T>G, rs6768972 and c.-59g>A, rs1672753 were selected based on MAF (minor allele frequency, 28% and 39% respectively), data from literature and location in gene’s regulatory region with potential influence on expression or activity of the encoded protein. Apart from SNP’s we chose to analyze other factors associated with disease’s activity, which can potentially affect its course.

## RESULTS

The distribution of the examined SNPs of *CRBN* gene (rs1672753, rs6768972) was not significantly correlated with such factors as: sex, age, family history of cancer, history of other cancers, type of monoclonal protein, class of light chains, disease stage and patient performance status. In patients diagnosed with secreting MM, TT genotype of the examined gene occurred more frequently than others (rs1672753: 51.7%, p=0.0025). In patients without t(4:16) translocation genotype AA (rs6768972: 55.6%, p=0.0030) was significantly more prevalent. Among patients exposed to carcinogens, the AA (rs6768972: 72%, p=0,0218) and TT (rs1672753: 80%, p=0.0150) genotypes were more prevalent. Genotypes AA, AC and CC of the *CRBN* gene (rs6768972) occurred respectively in 50%, 42.6% and 7.4% of subjects, whereas genotypes CC, CT and TT (rs1672753) occurred in 7.4%, 35.3% and 57.3% of patients (Table 1). Genotypes of both examined SNPs were within the Hardy-Weinberg equilibrium (rs6768972: p=0.7256 and rs1672753: p=0.6276).

**Table 1:** Patient characteristics with reference to the occurrence of *CRBN* gene genotypes (rs6768972 and rs1672753)

Variable	*CRBN* n (%)							
	rs6768972			*p* *C*	rs1672753			*P* *C*
	AA 34 (50)	AC 29 (42,6)	CC 5 (7,4)		CC 5 (7,4)	CT 24 (35,3)	TT 39 (57,3)	
**Sex**								
Men	21 (53,8)	15 (38,5)	3 (7,7)	0,7187	2 (5,6)	13 (33,3)	24 (61,5)	0,6082
Women	13 (44,8)	14 (48,3)	2 (6,9)	0,0981	3 (10,3)	11 (37,9)	15 (51,7)	0,120
**Age**								
<65	25 (54,4)	18 (39,1)	3 (6,5)	0,5817	4 (8,7)	15 (32,6)	27 (58,7)	0,7104
≥65	9 (40,9)	11 (50)	2 (9,1)	0,1250	1 (4,5)	9 (40,9)	12 (54,5)	0,0998
**Smoking**								
Non-smokers	23 (50)	19 (41,3)	4 (8,7)	0,6368	5 (10,9)	15 (32,6)	26 (56,5)	0,6082
Active smokers	5 (41,7)	7 (58,3)	-	0,190	-	5 (41,7)	7 (58,3)	0,196
Ex-smokers	6 (60)	3 (30)	1 (10)		-	4 (40)	6 (60)	
**Exposure to carcinogenic factors**								
Yes	18 (72)	6 (24)	1 (4)	**0,0218**	1 (4)	4 (16)	20 (80)	**0,0150**
No	16 (37,2)	23 (53,5)	4 (9,3)	**0,318**	4 (9,3)	20 (46,5)	19 (44,2)	**0,32**
**Family history of cancer**								
Yes	14 (48,3)	11 (37,9)	4 (13,8)	0,2074	4 (13,8)	10 (34,5)	15 (51,7)	0,2079
No	20 (51,2)	18 (46,2)	1 (2,6)	0,21	1 (2,6)	14 (35,9)	24 (61,5)	0,210
**History of other cancers**								
Yes	2 (66,7)	1 (33,3)	-	0,7910	-	1 (33,3)	2 (66,7)	0,8686
No	32 (49,2)	28 (43,1)	5 (7,7)	0,0828	5 (7,7)	23 (35,4)	37 (56,9)	0,0642
**Diagnosis**								
Secretory	25 (43,1)	28 (48,3)	5 (8,6)	0,0753	4 (6,9)	24 (41,4)	30 (51,7)	**0,0025**
Light chain disease	8 (100)	-	-	0,380	-	-	8 (100)	**0,479**
Non-secretory/plasmablastic	-	1 (100)	-		1 (100)	-	-	
Non-secretory/pasmacytoma	1 (100)	-	-		-	-	1 (100)	
**Monoclonal protein class**								
IgA	9 (52,9)	7 (41,2)	1 (5,9)	0,6655	1 (5,9)	7 (41,2)	9 (52,9)	0,9848
IgG	17 (41,5)	21 (51,2)	4 (9,8)	0,117	3 (7,3)	17 (41,5)	22 (53,7)	0,0227
**Light chain type**								
Lambda	15 (55,6)	9 (33,3)	3 (11,1)	0,3817	2 (7,4)	10 (37)	15 (55,6)	0,9163
Kappa	18 (46,2)	19 (48,7)	2 (5,1)	0,168	2 (5,1)	14 (35,9)	23 (59)	0,051
**Durie-Salmon stage**								
I	2 (50)	1 (25)	1 (25)	0,5681	1 (25)	1 (25)	2 (50)	0,4491
II	3 (42,9)	4 (57,1)	-	0,204	-	4 (57,1)	3 (42,9)	0,227

III	29 (50,9)	24 (42,1)	4 (7)		4 (7)	19 (33,3)	34 (59,7)	
**ISS stage**								
1	5 (33,3)	7 (46,7)	3 (20)	0,2882	3 (20)	5 (33,3)	7 (46,7)	0,3621
2	12 (54,5)	9 (40,9)	1 (4,54)	0,265	1 (4,5)	8 (36,4)	13 (59,1)	0,248
3	15 (51,7)	13 (79,3)	1 (3,4)		1 (3,4)	11 (37,9)	17 (58,6)	
**Deletion17p**								
Present	5 (55,6)	3 (33,3)	1 (11,1)	0,9044	-	4 (44,4)	5 (55,6)	0,6760
Absent	15 (53,6)	11 (39,3)	2 (7,1)	0,0735	2 (7,2)	10 (35,7)	16 (57,1)	0,144
**Translocation t(4;14)**								
Present	2 (33,3)	4 (66,7)	-	0,2563	-	4 (66,7)	2 (33,3)	0,2655
Absent	18 (58,1)	10 (32,2)	3 (9,7)	0,262	2 (6,5)	10 (32,2)	19 (61,3)	0,259
**Translocation t(4;16)**								
Present	-	-	1 (100)	**0,0030**	-	1 (100)	-	0,4299
Absent	20 (55,6)	14 (38,9)	2 (5,6)	**0,489**	2 (5,6)	13 (36,1)	21 (58,3)	0,209
**Renal function**								
A	29 (50,9)	23 (40,4)	5 (8,7)	0,4832	5 (8,7)	19 (33,4)	33 (57,9)	0,5049
B	5 (45,5)	6 (54,5)	-	0,145	-	5 (45,5)	6 (54,5)	0,140
**Stage of chronic kidney disease**								
G1	15 (46,9)	15 (46,9)	2 (6,2)	0,8964	3 (9,3)	11 (34,3)	18 (59,4)	0,8797
G2	8 (53,3)	5 (33,3)	2 (13,4)	0,260	2 (13,4)	4 (26,6)	9 (60)	0,266
G3a	3 (60)	1 (20)	1 (20)		-	2 (40)	3 (60)	
G3b	3 (50)	3 (50)	-		-	3 (50)	3 (50)	
G4	3 (60)	2 (40)	-		-	1 (20)	4 (80)	
G5	2 (40)	3 (60)	-		-	3 (60)	2 (40)	
**Performance status**								
0	5 (50)	2 (20)	3 (30)	0,0719	3 (30)	2 (20)	5 (50)	0,6871
1	9 (45)	9 (45)	2 (10)	0,382	1 (5)	8 (40)	11 (55)	0,204
2	15 (51,7)	14 (48,3)	-		-	12 (41,4)	17 (59,6)	
3	5 (55,6)	4 (44,4)	-		1 (11,1)	2 (22,2)	6 (66,7)	
**Body weight loss before treatment**								
Yes	17 (51,5)	15 (45,5)	1 (3)	0,4112	2 (5,7)	12 (34,3)	19 (54,3)	0,9199
No	17 (48,6)	14 (40)	4 (11,4)	0,160	3 (9)	12 (30,3)	20 (60,6)	0,0495
5%	7 (53,8)	5 (38,5)	1 (7,7)	0,4082	1 (7,7)	4 (30,8)	8 (61,5)	0,8448
10%	10 (50)	10 (50)	-	0,227	1 (5)	8 (40)	11 (55)	0,101
**Anemia grade before treatment (according to WHO)**								
Absent	13 (59,1)	8 (36,4)	1 (4,5)	0,1334	1 (4,5)	7 (31,8)	14 (63,4)	0,7465
I (mild)	6 (30)	10 (50)	4 (20)	0,393	3 (15)	9 (45)	8 (40)	0,264
II (moderate)	11 (61,1)	7 (38,9)	-		1 (5,6)	5 (27,8)	12 (66,7)	
III (severe)	4 (66,7)	2 (33,3)	-		-	2 (33,3)	4 (66,7)	
IV (life-threatening)	-	2 (100)	-		-	1 (50)	1 (50)	

Median number of treatment cycles was 6 (range: 1-12), while the follow-up period duration averaged 27 months (range: 2-130). Median PFS was 15 months and OS 79 months. Almost half of the patients (47.1%) were subjected to autologous hematopoietic stem cell transplantation (auto-HSCT). Median time to auto-HSCT was 12 months (range: 1-36 months). Auto-HSCT was associated with significant extension of PFS (23 vs 10 months; HR=0.55, 95% CI: 0.30-0.99, p=0.0210) and OS (not reached vs 50 months; HR=0.30, 95% CI: 0.13-0.70, p=0.0039).

**Figure 1:**
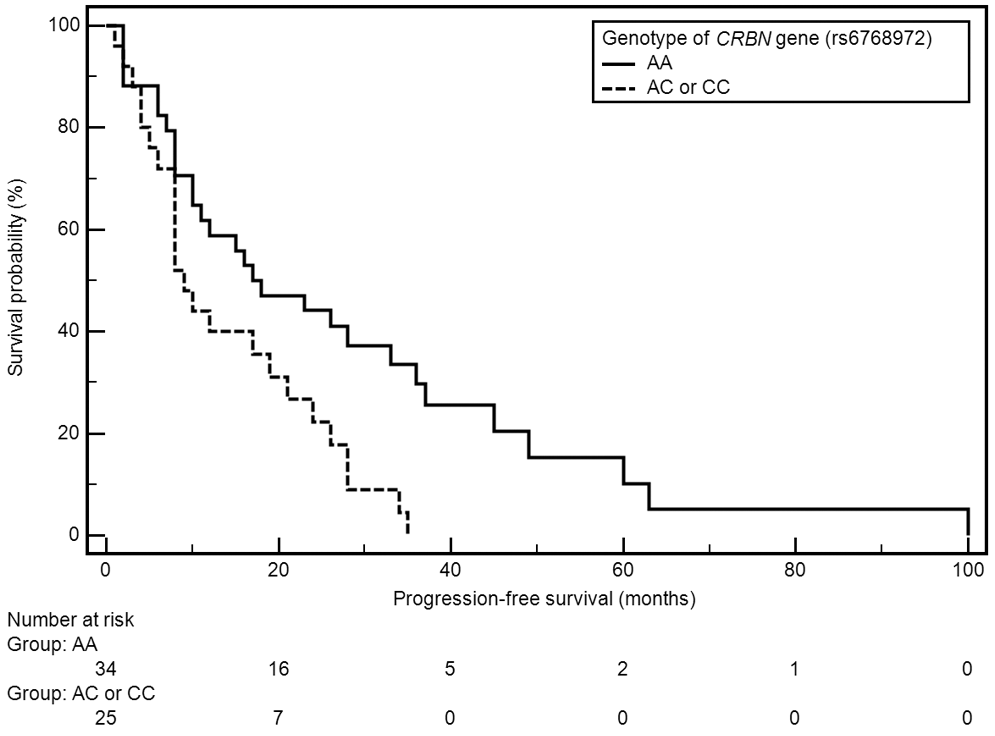
Kaplan-Meier curves illustrating PFS differences between genotype AA and AC, CC of *CRBN* gene (rs6768972)

**Figure 2:**
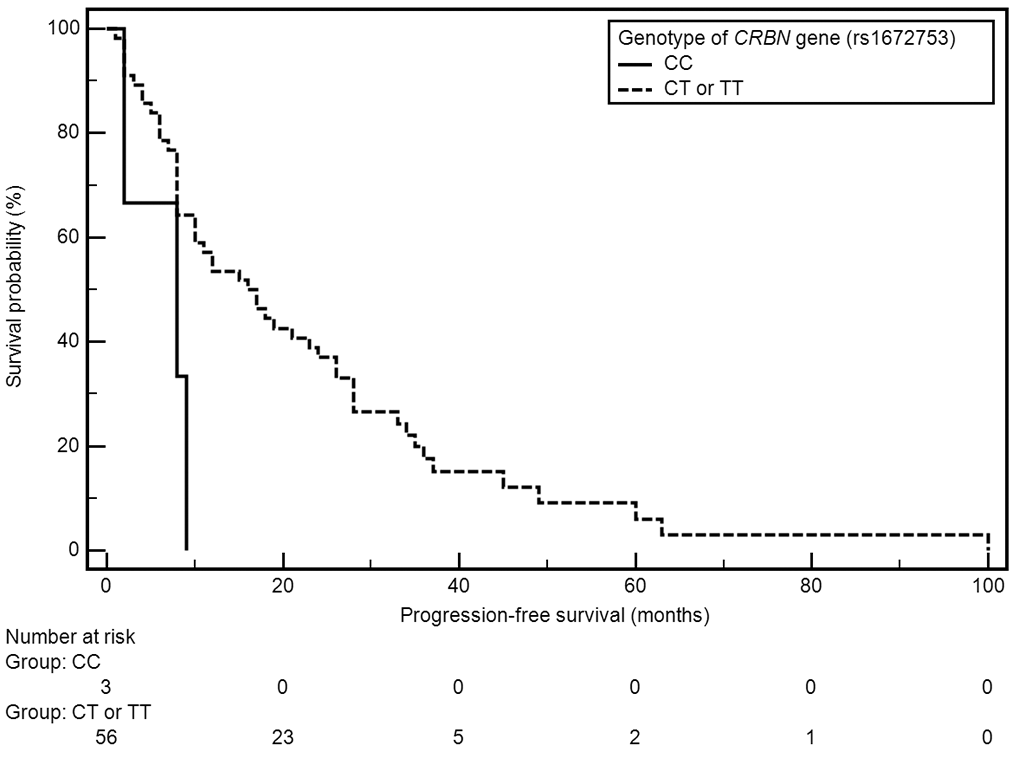
Kaplan-Meier curves illustrating PFS differences between genotype CC and CT, TT of *CRBN* gene (rs1672753)

Factors with significant impact on median PFS included grade of anemia before treatment and presence of both examined polymorphic variants of *CRBN* gene (Table 2). A significant shortening of median PFS was observed in patients with anemia grade ≥ II^o^ according to WHO (21 vs 8 months; HR=0.49, 95% CI: 0.26-0.93, p=0.0086). Shorter median PFS was also observed in carriers of genotypes CC (rs1672753) of *CRBN* gene (4 vs 16 months; HR=3.93, 95% CI: 0.26-59.64, p=0.0321), whereas the presence of genotype AA (rs6768972) was associated with significant PFS extension (median 18 vs 9 months; HR=0,49, 95% CI: 0.26-0.91, p=0.0062) (Figures 1, 2).

**Table 2:** The influence of selected factors on the risk of PFS and OS shortening in multivariate analysis (Cox logistic regression model) (model adjustment: p=0,0007; χ^2^=41,88)

Variable	Progression free survival				Overall survival			
	β	p	HR	95% CI	β	P	HR	95%CI
**Sex**								
Male	1,5240	**0,0179**	**4,5905**	**1,3085-16,1046**	1,0058	0,3945	2,7341	0,2732 - 27,3656
**Age**								
≥65	-0,3473	0,6036	0,7066	0,1918-2,6033	-0,2613	0,8237	0,7701	0,0782 - 7,5811
**Monoclonal protein class**								
IgA	-0,6975	0,2284	0,4978	0,1609-1,5398	-1,4846	0,1460	0,2266	0,0309 - 1,6602
**Light chain type**								
Kappa	-0,2881	0,6230	0,7497	0,2390-2,3511	0,7021	0,3740	2,0180	0,4327 - 9,411
**ISS stage**								
1 2	-0,3673	0,6728	0,6926	0,1270-3,7762	-12,0997	0,9659	0,0000	1,0277E-245 - 3,00924E+234
**Renal function**								
A	-2,5102	**0,0294**	**0,0812**	**0,0086-0,7687**	-1,7350	0,3109	0,1764	0,0063 - 4,9704
**Body weight loss before treatment**								
Yes	1,1288	**0,0291**	**3,0920**	**1,1273-8,4806**	0,1953	0,8403	1,2156	0,1837 - 8,0453
**The degree of anemia before treatment (according to WHO)**								
Absent or I^o^	-1,4265	**0,0285**	**0,2401**	**0,0674-0,8551**	-0,6095	0,4688	0,5436	0,1054 - 2,8038
**ALB**								
Low	-0,1431	0,8201	0,8667	0,2542-2,9554	-0,2914	0,7595	0,7472	0,1168 - 4,7803
**CRP**								
High	0,4095	0,4092	1,5060	0,5723-3,9632	2,0013	0,0646	7,3990	0,8955 - 61,1308
**B2M**								
High	-1,7173	**0,0080**	**0,1795**	**0,0508-0,6347**	-0,5284	0,5927	0,5896	0,0859 - 4,0458
**Supportive care**								
Yes	-1,4846	0,0840	0,2266	0,0424-1,2105	-2,3265	0,1445	0,0976	0,0044 - 2,1868
**Auto HSCT**								
No	-1,5781	**0,0052**	**0,2064**	**0,0687-0,6202**	-1,5363	0,1415	0,2152	0,0280 - 1,6510
**Thrombocytopenia**								
Absent	-2,7610	**0,0007**	**0,0632**	**0,0129-0,3096**	-2,5881	**0,0090**	**0,0752**	**0,0109 - 0,5194**
**Lymphopenia**								
2-4^o^	1,2439	0,0555	3,4689	0,9773-12,3132	-1,5220	**0,0486**	**0,2183**	**0,0485 - 0,9830**
**Genotype CRBN (rs6768972)**								
AC or CC	2,3559	**0,0004**	**10,5477**	**2,9025-38,3297**	-0,2967	0,7415	0,7433	0,1287 - 4,2928
**Genotype CRBN (rs1672753)**								
CT or TT	-2,8528	**0,0442**	**0,0577**	**0,0036-0,9153**	11,0290	0,9766	61638,7219	0,0000 - 10,1423E+303

A series of associations has been demonstrated between the analyzed factors and patients OS (Table 3). Patients in ISS stage I had significantly longer median OS compared to the rest of the subjects (82 vs 38 months; HR=0.42, 95% CI: 0.18-0.99; p=0.0338). In patients with normal renal function, classified as category A according to Durie-Salmon scale, significant extension of OS was also observed (82 vs 33 months; HR=0.34, 95% CI: 0.09-1.27; p=0.0136). Clinical factors associated with significant shortening of OS included: weight loss before treatment (56 vs 103 months; HR=3.22, 95% CI: 1.42-7.77; p=0.0028) and presence of anemia grade ≥ II^o^ (38 vs 103 months, respectively; HR=0.24, 95% CI: 0.09-0.60; p=0.0002). Male sex was associated with insignificantly higher risk of OS shortening (50 vs 79 months; HR=2.45, 95% CI: 1.06-5.63; p=0.0585).

**Table 3:** Median PFS and OS of MM patients depending on selected factors

Variable	Progression free survival		Overall survival	
	Median (months)	p HR (95% CI)	Median (months)	p HR (95% CI)
	15		79	
**Sex**				
Men	12	0,6938	50	0,0585
Women	15	0,89 (0,50-1,61)	79	2,45 (1,06-5,63)
**Age**				
<65	15	0,5942	82	0,2853
≥65	17	0,85 (0,44-1,64)	38	0,63 (0,23-1,70)
**Smoking**				
Non-smokers	12	0,8644	79	0,6341
Active and ex-smokers	15	0,95 (0,52-1,73)	103	1,23 (0,50-2,99)
**Exposure to carcinogenic factors**				
Yes	21	0,7515	79	0,5778
No	10	0,91 (0,51-1,63)	-	1,25 (0,55-2,86)
**Family history of cancer**				
Yes	19	0,5949	103	0,0662
No	10	0,86 (0,49-1,52)	50	0,45 (0,20-1,02)
**History of other cancers**				
Yes	11	0,8036	79	0,7547
No	15	1,19 (0,26-5,51)	82	0,73 (0,13-4,17)
**Diagnosis**				
Secretory				
Light chain disease	15		79	
Non-secretory/plasmablastic type	11	0,7947	103	0,7487
Non-secretory/plasmacytoma		1,10 (0,54-2,22)		0,85 (0,30-2,41)
**Monoclonal protein class**				
IgA	17	0,2375	38	0,4391
IgG	12	0,69 (0,37-1,31)	79	1,43 (0,53-3,85)
**Light chain type**				
Lambda	11	0,2281	56	0,1122
Kappa	21	1,40 (0,75-2,61)	103	1,90 (0,79-4,57)
**Durie-Salmon stage**				
I, II	7	0,7786	82	0,6610
III	16	1,17 (0,33-4,12)	79	1,24 (0,43-3,58)

**ISS stage**				
1, 2	24	0,2258	82	**0,0338**
3	11	0,62 (0,31-1,25)	38	**0,42 (0,18-0,99)**
**Deletion 17p**				
Present	10	0,7788	-	0,8796
Absent	10	0,89 (0,37-2,09)	50	1,10 (0,29-4,15)
**Translocation t(4;14)**				
Present	5	0,0619	33	**0,0453**
Absent	12	2,28 (0,69-7,56)	56	**2,99 (0,59-15,10)**
**Translocation t(4;16)**				
Present	9	0,5061	-	0,6176
Absent	12	1,91 (0,13-28,67)	50	-
**Renal function**				
A	17	0,1250	82	**0,0136**
B	8	0,55 (0,20-1,52)	33	**0,34 (0,09-1,27)**
**Stage of chronic kidney disease**				
G1/G2	18	0,5056	82	0,0572
G3a/G3b/G4/G5D	8	0,83 (0,44-1,56)	38	0,46 (0,17-1,22)
**Performance status**				
0, 1	12	0,7900	82	0,1958
2, 3	16	1,08 (0,61-1,90)	79	0,58 (0,26-1,33)
**Body weight loss before treatment**				
Yes	10	0,1082	56	**0,0028**
No	18	1,55 (0,86-2,81)	103	**3,32 (1,42-7,77)**
**Anemia grade before treatment (WHO)**				
Absent or I^o^	21	**0,0086**	103	**0,0002**
II^o^, III^o^ or IV^o^	8	**0,49 (0,26-0,93)**	38	**0,24 (0,09-0,60)**
**HGB**				
Normal	37	**0,0235**	103	0,1238
Low	10	**0,40 (0,21-0,76)**	79	0,35 (0,13-0,94)
**PLT**				
Normal	17	0,2227	103	**0,0306**
Low	10	0,68 (0,34-1,38)	38	**0,42 (0,15-1,13)**
**ALB**				
Normal	21	0,1835	103	**0,0237**
Low	9	0,70 (0,39-1,24)	56	**0,40 (0,17-0,93)**

**CRP**				
Normal	18	0,7406	82	**0,0456**
High	11	0,91 (0,50-1,66)	38	**0,44 (0,18-1,06)**
**LDH**				
Normal	12	0,9448	79	0,3383
High	16	0,97 (0,38-2,49)	-	2,52 (0,64-9,85)
**Calcium**				
Normal	17	0,7698	79	0,7630
High	8	1,09 (0,58-2,07)	-	0,87 (0,33-2,28)
**B2M**				
Normal	21	0,1327	82	**0,0180**
High	10	0,66 (0,36-1,19)	38	**0,37 (0,16-0,88)**
**Creatinine**				
Normal	19	0,3620	82	0,0617
High	9	0,77 (0,42-1,42)	56	0,47 (0,19-1,15)
**eGFR**				
Normal	18	0,7928	82	0,1897
Low	10	1,07 (0,61-1,89)	56	0,59 (0,26-1,34)
**Genotype CRBN (rs6768972)**				
AA	18	**0,0062**	82	0,7984
AC or CC	9	**0,49 (0,26-0,91)**	-	0,90 (0,39-2,07)
**Genotype CRBN (rs6768972)**				
AC	10	**0,0179**	38	0,2847
AA or CC	17	**1,89 (1,00-3,56)**	82	1,52 (0,64-3,62)
**Genotype CRBN (rs6768972)**				
CC	8	0,1812	-	0,1682
AA or AC	16	2,42 (0,28-20,74)	79	-
**Genotype CRBN (rs1672753)**				
CC	4	**0,0321**	-	0,5579
CT or TT	16	**3,93 (0,26-59,64)**	79	0,56 (0,12-2,63)
**Genotype CRBN (rs1672753)**				
CT	15	0,7318	103	0,5232
CC or TT	12	1,10 (0,62-1,95)	82	1,30 (0,57-2,96)
**Genotype CRBN (rs1672753)**				
TT	16	0,4248	79	0,7397
CC or CT	12	0,79 (0,45-1,41)	103	0,87 (0,37-2,01)

Among the analyzed cytogenetic abnormalities, the presence of translocation t(4;14) was associated with significant OS shortening (33 vs 56 months; HR=2.99, 95% CI: 0.59-15.10; 0.0453). Significant OS extension was observed in patients with normal platelet count (103 vs 38 months; HR=0.42, 95% CI: 0.15-1.13; p=0.0306), albumin (103 vs 56 months; HR=0.40, 95% CI: 0.1793; p=0.0237), CRP (82 vs 38 months; HR=0.44, 95% CI: 0.18-1.06; p=0.0456) and β2-microglobulin (B2M) (not reached vs 38 months; HR=0.37, 95% CI: 0.16-0.88; p=00180). None of the variants of examined SNPs affected the risk of OS shortening.

Multivariate analysis using Cox’s regression model revealed factors which were independently associated with significantly higher risk of PFS shortening: male sex (p=0.0179; HR=4.59, 95%CI: 1.31-16.10), weight loss before treatment (p=0.0291; HR=3.09, 95% CI: 1.13-8.48) and the presence of genotypes AC or CC (rs6768972, p=0.0004; HR=10.55, 95% CI: 2.90-38.33). Factors which were independently associated with significantly lower risk of PFS shortening included: normal renal function (p=0.0294; HR=0.08, 95%CI: 0.01-0.77), absence of anemia or grade I anemia before treatment (p=0.0285; HR=0.24, 95%CI: 0.07-0.85), normal B2M level (p=0.0080; HR=0.18, 95%CI: 0.05-0.63), auto-HSCT (p=0.0052; HR=0.21, 95% CI: 0.07-0.62), absence of thrombocytopenia in the course of treatment (p=0.0007; HR=0.06, 95%: 0.01-0.31) and the presence of genotypes CT or TT (rs1672753, p=0.0442; HR=0.06, 95% CI: 0.01-0.91). Factors which were independently associated with significantly lower risk of OS shortening included absence of thrombocytopenia (p=0.0090; HR=0.07, 95%CI: 0.01-0.52) and absence of lymphopenia or grade I lymphopeniaaccording to CTCAE (p=0.0486; HR=0.22, 95%CI: 0.05-0.98) (Table 2).

## DISCUSSION

The goal of the first line MM treatment is to achieve the best possible response - preferably remission. Until recently, CTD chemotherapy was a standard in newly diagnosed MM patients who qualify for high-dose chemotherapy supported by auto-HSCT. This approach was also commonly used for treatment of elderly patients. Currently, with the increasing availability of bortezomibe and lenalidomide, its use has been limited. It is estimated that CTD therapy combined with auto-HSCT objective responses rate (ORR) reaches 82.5% with PFS of 34. Patients who do not qualify for transplantation reach ORR of 63.8%, PFS 13 months and OS 33.2 months [12, 13, 14, 15]. Unfortunately, not all patients benefit from treatment, and the therapy is often associated with adverse effects including thromboembolism, peripheral neuropathy, thrombocytopenia, neutropenia, constipation, somnolence, urticarial skin lesions, Stevens-Johnson syndrome, arrhythmia and hypothyroidism. As with many other cancers, there is a correlation between the extent of response and the OS of patients [16, 17]. It is therefore critical to identify factors that would allow for appropriate patients stratification for the selected treatment regimen.

Anemia is an independent adverse prognostic factor both for patients treated with conventional chemotherapy and novel agents (thalidomide, bortezomibe or lenalidomide). Our results show that the diagnosis of anemia ≥ II^0^ (WHO) before treatment is associated with significantly shorter PFS (8 vs 21 months, p = 0.0086) and OS (38 vs 108 months, p = 0.0002). Those results are consistent with many previous reports. MM patients with hemoglobin concentration ≤ 10g/dl achieve shorter survival, the difference in OS is currently at 34 vs 70 months [18, 19].

Renal impairment in patients treated with traditional chemotherapy was associated with poor prognosis, high incidence of early deaths and median survival shorter than 2 years [20, 21]. Introduction of new drugs in the treatment of MM allowed for significant improvement of treatment outcomes. A significant OS elongation from 20.5 to 31 months (p = 0.03) was obtained, but this is still a significantly lower result than in non-compromised patients [18, 22]. Jung et al. demonstrated that even now, in the era of wide availability of novel agents, serum creatinine concentration ≥ 2 mg/dL is an independent risk factor (p = 0.015) of early mortality. Out of 542 subjects, 66.1% (n = 358) were treated with first line regimens containing thalidomide (usually CTD or MPT) [23]. In our study we confirmed that serum creatinine ≥ 2 mg/dL adversely affects prognosis and is associated with significantly shorter OS (82 vs 33 months, p = 0.0136) in MM patients.

Another confirmed factor affecting the prognosis of MM patients is the stage of the disease. Of the two currently used scales: Durie-Salmon’s and ISS (International Staging System) the later has a definite advantage. It is a simple risk stratification algorithm based on two laboratory parameters. The goal of the authors of the scale was to reflect the tumor mass and degree of renal involvement by measuring serum β2-microglobulin concentration and indirectly analyzing bone marrow microenvironment via albumin concentration, which is closely associated with IL-6 concentration. According to the ISS, we distinguish three stages of MM with different prognosis. The median OS in subsequent stages of the disease was determined as 62, 44 and 29 months [24]. In our study, a statistically significant difference in OS between patients in ISS stage I and those classified to higher stages (82 vs 38 months, p = 0.0338) was observed. In the group of patients with normal β2-microglobulin and albumin concentration a significant increase in OS was observed (respectively, not reached vs 38 months, p = 0.0018 and 103 vs 56 months, p = 0.0237).

The age of patients at the time of diagnosis is also important in predicting the course of MM. Due to non-haematological complications, especially severe infections and cardiological complications, elderly patients relatively more often require dose reductions, or even withdrawal of treatment which directly translates into its outcome. Bringhen et al. in their meta-analysis of 1435 elderly MM patients treated with regimens containing thalidomide and/or bortezomib determined the median OS to be 50 months. Authors demonstrated that patients older than 75 (p <0.001), with renal failure (p <0.001), serious infectious, cardiac or gastroenterological complications (p <0.001) and whose treatment was stopped due to adverse effects (p = 0.01) had significantly higher risk of death. They also pointed out that intensive treatment regimens containing combinations of bortezomib and thalidomide are poorly tolerated by older patients and appear to be a wrong therapeutic option for them [25]. Our results, similarly to those presented in previous studies, prove that CTD is effective not only in younger patients qualifying for autoHSCT but also in elderly [14, 15]. In our study there were no significant correlations between the age of patients and response to treatment or prognosis.

It was demonstrated that male sex was an independent prognostic factor associated with significantly higher risk of PFS shortening (p = 0.0179; HR = 4.59, 95% CI: 1.31-16.10). These results differ from previous observations. In MRC Myeloma IX study, Boyd et al., analyzed the presence of molecular disorders and the response to treatment in reference to the sex of the studied patients. The authors showed that women were more likely to have adverse genetic aberrations and that female gender was associated with significantly shorter OS (44.8 months for women vs 49.9 months for men, p = 0.02). The relatively small number of patients in our study in comparison to Myeloma IX study (n = 68 vs 1960) makes it difficult to interpret the significance of our results [26].

Cytogenetic abnormalities affect efficacy of CTD chemotherapy; the presence of translocation t(4;14) was associated with poor response to treatment and shorter OS (33 vs 56 months, p <0.0453). Similar conclusions were presented by Chang et al. In a study evaluating the efficacy of CTD in patients with MM prior to planned auto-HSCT, authors demonstrated that the most important factor associated with shorter PFS and OS (p <0.05) was the presence of t(4;14) translocation. Thus, they suggested that the CTD regimen might not be optimal therapeutic option for this group of patients and that regimens containing bortezomibe should be used in the first line of treatment [27].

Beason et al. demonstrated that underweight patients (BMI <18.5 kg/m2) have higher mortality when compared to patients with normal or above normal BMI. Researchers observed significant differences in median OS of patients depending on individual BMI categories - 14.3 months in the group of underweight patients, 23.7 months in subjects with normal body weight and 31.7 months in overweight and obese. They also proved that an unintentional weight loss of ≥10% observed during one year prior to the diagnosis is an important risk factor (HR: 1.52, 95% CI: 1.34-1.72) [28]. Our study used clinical data obtained from medical history. Patients with significant weight loss before treatment had significantly shorter OS compared to other patients (56 vs 103 months, p = 0.0028).

Among the standard laboratory parameters analyzed in the performed work, the concentration of CRP influenced the survival of patients. Median OS in the group of subjects with elevated values was significantly shorter compared to the remaining patients (38 vs 82 months, p = 0.0456). CRP is a nonspecific exponent of the inflammatory process, also caused by cancer. The results of previous studies suggest that in myeloma CRP concentration is dependent on IL-6 activity and indirectly reflects the state of bone marrow microenvironment. Singhal et al. in the very first study with thalidomide proved that elevated CRP level is one of the factors associated with significantly shorter ESF (p = 0.007) [29]. These observations were confirmed in later publications [30]. Barlogie et al. concluded that high CRP concentration is one of the hallmarks of highly aggressive disease [31].

Thrombocytopenia in patients with MM is an unfavorable prognostic factor. It indirectly reflects the degree of bone marrow involvement, and may also result from hypersecretion of proinflammatory cytokines that inhibit the maturation of megakaryocytes and interfere with effective thrombopoesis [32]. Is is associated with shorter OS in patients with MM [33]. Jung et al. showed that thrombocytopenia is also a risk factor of early mortality [23]. In our study, a group of patients with decreased PLT level had a significantly shorter median OS compared to the remaining patients (38 vs 103 months, p = 0.0306). The occurrence of thrombocytopenia in the course of treatment was also associated with shorter OS (33 vs 82 months, p = 0.0065).

Huang et al. showed that high expression of cereblon in malignant plasma cells was associated with better clinical response in patients treated with thalidomide and lenalidomide. Newly diagnosed CRBN(+) patients treated with TD regimen had significantly higher response rate compared to CRBN(-) patients (75% vs. 29%, p=0.005). No correlation was found in patients treated with regimen containing bortezomibe [34]. Jung et al. compared the efficacy of thalidomide and bortezomibe in the treatment of older patients with known cereblon expression. In the CRBN(+) group, a better clinical response to thalidomide based chemotherapy was observed compared to patients with no CRBN expression (CR 35.0 vs 11.8%; HR = 4.038, p = 0.137). Furthermore, in CRBN(+) patients, no significant differences in survival was observed irrespective of the treatment regimens containing thalidomide or bortezomibe. The time to progression (TTP) were 13.8 vs 15.6 months (p = 0.842), while OS was 39.3 vs. 30.1 months (p = 0.074) respectively. Researchers suggested that in the CRBN(+) group of patients the efficacy of both drugs is comparable, and that CRBN expression status evaluation was very useful in the selection of treatment in elderly patients [35].

In our study, two SNPs of the *CRBN* gene (rs6768972 and rs1672753) were analyzed. Both examined SNPs significantly affected patients PFS. None of the examined SNPs had a significant effect on the OS in the study group. Single report of similar results has been presented at the 56^th^ ASH Conference (2014) describing 169 MM patients with resistant and recurrent disease which have been treated with lenalidomide based regimen. The patients were evaluated for the effect of 14 selected SNPs located in the *CRBN* gene coding region. Two of the polymorphisms (rs1714327G>C and rs1705814T>C) were associated with significantly lower probability of response to treatment ≥ PR (OR = 0.25, 95% CI 0.10-0.63; p = 0.0033 and OR = 0.21, 95% CI 0.07- 0.61; p = 0.0041). In addition, one of these SNPs, namely rs1705814T>C, was also associated with shorter PFS (OR = 2.49; 95% CI 1.31-4.74; p = 0.0054) [36]. Butrym et al. analyzed two selected SNPs found in the non-coding regions of the *CRBN* gene (rs711613, rs1045433) in the context of response to treatment in patients diagnosed with MM. The authors demonstrated that, in the case of one of the SNP’s (rs711613), the occurrence of allele A was associated with significantly better clinical response (p = 0.012). In the group of patients treated with CTD chemotherapy, however, no relationship was found between the studied polymorphisms and the efficacy of treatment [37].

With multiple new drugs available, selection of the first line treatment in MM patients requires careful analysis of all available predictive and prognostic factors. Based on the results of our study, since 75% of patients achieved objective response it can be concluded that CTD chemotherapy is still an effective therapeutic option. In the group of patients with high-risk MM, i.e. with advanced stage of disease according to ISS, severe anemia, chronic kidney disease, weight loss and the presence of t(4; 14) translocation the efficacy of CTD chemotherapy is significantly lower.

The SNPs of *CRBN* gene *(rs6768972, rs1672753)* proved to be a significant, independent predictive factors of the efficiency of CTD regimen in the study group. The presence of genotype AA (rs6768972) of *CRBN* gene was correlated with the extension of median PFS. The presence of AC (rs6768972) and CC (rs1672753) variants of the *CRBN* gene was associated with significant shortening of PFS. The presence of *CRBN* gene SNPs may be useful in qualifying patients for treatment with thalidomide containing regimens.

## MATERIALS AND METHODS

The study group included 68 patients with MM, who were subjected to first line CTD therapy in 28-day regimens with standard doses: thalidomide 100 mg/day p.o, cyclophosphamide 300-500 mg/week p.o. and dexamethasone 10-20 mg/day p.o. on days 1-4 and 8-11. MM diagnosis was based on SLiM CRAB criteria, disease staging was determined according to Durie-Salmon and ISS staging systems. Patients performance status was evaluated using ECOG-WHO scale. Treatment response was assessed according to IMWG 2016 criteria. The severity of adverse effects was evaluated using CTCAE (version 4.03). The detailed patient characteristics are presented in Table 4.

**Table 4:** Demographic and clinical characteristics of the study group

Variable	n (%)
**Sex**	
Men	39 (57.4)
Women	29 (42.6)
**Age**	
Median	62
Average ± standard deviation	62.21 ± 11.01
Scope	39.-87
Diagnosis	
Secretory	58 (85.2)
Light chain disease	8 (11.8)
Non-secretory/plasmablastic	1 (1.5)
Non-secretory/plasmacytoma	1 (1.5)
**Monoclonal protein class**	
IgA	17 (29.3)
IgG	41 (70.7)
**Light chain type**	
Lambda	27 (40.9)
Kappa	39 (59.1)
**Durie-Salmon stage**	
I	4 (5.9)
II	7 (10.3)
III	57 (83.8)
**ISS stage**	
1	15 (22.8)
2	22 (33.3)
3	29 (43.9)
**Percentage of plasma cells in bone marrow (%)**	
median	30
average ± standard deviation	32 ± 21
scope	1-90
**Deletion 17p**	
Present	9 (13.2)
Absent	28 (41.2)
No materials	31 (45.6)
**Translocation t(4;14)**	
Present	6 (8.8)
Absent	31 (45.6)
No materials	31 (45.6)
**Translocation t(4;16)**	
Present	1 (1.5)
Absent	36 (52.9)
No materials	31 (45.6)
**Renal function**	
A	57 (83.8)
B	11(16.2)
**Stage of chronic kidney disease**	
G1	32 (47)
G2	15 (22)
G3a	5 (7.4)
G3b	6 (8.8)
G4	5 (7.4)
G5D	5 (7.4)
**Performance status**	
0	10 (14.7)
1	20 (29.4)
2	29 (42.7)
3	9 (13.2)
**Body weight loss before treatment**	
Yes	33 (48.5)
No	35 (51.5)
5%	13 (39.4)
10%	20 (60.6)
**Anemia grade before treatment (WHO)**	
Absent	22 (32.4)
I (mild)	20 (29.4)
II (moderate)	18 (26.5)
III (severe)	6 (8.8)
IV (life-threatening)	2 (2.9)

The examination of SNPs of the promoter region of CRBN gene (rs1672753 and rs6768972) was conducted using genetic material - DNA isolated from peripheral blood leukocytes. DNA was isolated using QIAamp DNA Blood Mini Kit (Qiagen). The measurement of the quality and quantity of the isolated DNA was performed using NanoDrop 2000 UV-Vis Spectrophotometer (ThermoFisher). The analysis of *CRBN* gene SNPs (rs1672753 and rs6768972) was performed using Real-Time PCR genotyping technique with specific Taqman probes (Life Technologies, USA). Statistical analysis of the results was conducted using MedCalc 15.8 and Statistica 10 software. Chi-square test (χ2) was used to evaluate Hardy-Weinberg (H-W) equilibrium, the correlation of various demographic and clinical factors with the distribution of polymorphic variants of *CRBN* gene and the influence of the studied factors on the probability of obtaining response to treatment (odds ratio). Kaplan-Meier estimation method and Cox’s logistic regression model were used to evaluate the probability of survival and the occurrence of progression depending on the distribution of examined factors.

## Abbreviations

CRBN: cereblon
CTD: cyclophosphamide, thalidomide, dexamethasone
IFN−γ: interferon γ
IL-2: interleukin 2
IMiDs: immunomodulatory drugs
MM: multiple myeloma
ORR: objective responses rate
OS: overall survival
PFS: progression free survival
SNP’s: single nucleotide polymorphisms

## References

[1] Kumar SK, Dispenzieri A, Lacy MQ, Gertz MA, Buadi FK, Pandey S, Kapoor P, Dingli D, Hayman SR, Leung N, Lust J, McCurdy A, Russell SJ (2014). Continued improvement in survival in multiple myeloma: changes in early mortality and outcomes in older patients. Leukemia.

[2] Siegel RL, Miller KD, Jemal A (2016). Cancer statistics, 2016. CA Cancer J Clin.

[3] Sze DM, Brown R, Yang S, Ho PJ, Gibson J, Joshua D (2006). The use of thalidomide in myeloma therapy as an effective anticancer drug. Curr Cancer Drug Targets.

[4] Fuchida SI, Shimazaki C, Hirai H, Akamatsu S, Yamada N, Uchida R, Okano A, Okamoto M, Inaba T, Taniwaki M (2008). The effects of thalidomide on chemotactic migration of multiple myeloma cell lines. Int J Lab Hematol.

[5] Ito T, Ando H, Handa H (2011). Teratogenic effects of thalidomide: molecular mechanisms. Cell Mol Life Sci.

[6] Fionda C, Abruzzese MP, Zingoni A, Cecere F, Vulpis E, Peruzzi G, Soriani A, Molfetta R, Paolini R, Ricciardi MR, Petrucci MT, Santoni A, Cippitelli M (2015). The IMiDs targets IKZF-1/3 and IRF4 as novel negative regulators of NK cell-activating ligands expression in multiple myeloma. Oncotarget.

[7] Broyl A, Kuiper R, van Duin M, van der Holt B, el Jarari L, Bertsch U, Zweegman S, Buijs A, Hose D, Lokhorst HM, Goldschmidt H, Sonneveld P, Dutch-Belgian HOVON group, German GMMG Group (2013). High cereblon expression is associated with better survival in patients with newly diagnosed multiple myeloma treated with thalidomide maintenance. Blood.

[8] Schuster SR, Kortuem KM, Zhu YX, Braggio E, Shi CX, Bruins LA, Schmidt JE, Ahmann G, Kumar S, Rajkumar SV, Mikhael J, Laplant B, Champion MD (2014). The clinical significance of cereblon expression in multiple myeloma. Leuk Res.

[9] Robert J, Morvan VL, Smith D, Pourquier P, Bonnet J (2005). Predicting drug response and toxicity based on gene polymorphisms. Crit Rev Oncol Hematol.

[10] Duffy MJ, Crown J (2008). A personalized approach to cancer treatment: how biomarkers can help. Clin Chem.

[11] Unger FT, Witte I, David KA (2015). Prediction of individual response to anticancer therapy: historical and future perspectives. Cell Mol Life Sci.

[12] Morgan GJ, Davies FE, Gregory WM, Bell SE, Szubert AJ, Navarro Coy N, Cook G, Feyler S, Johnson PR, Rudin C, Drayson MT, Owen RG, Ross FM (2012). Cyclophosphamide, thalidomide, and dexamethasone as induction therapy for newly diagnosed multiple myeloma patients destined for autologous stem-cell transplantation: MRC Myeloma IX randomized trial results. Haematologica.

[13] Dmoszynska A, Walter-Croneck A, Hus I, Grzasko N, Manko J, Jedrzejczak WW, Charlinski G, Usnarska-Zubkiewicz L, Skotnicki A, Wolska-Smolen T, Piszcz J, Kloczko J (2010). The efficacy and safety of the low-thalidomide dose CTD (cyclophosphamide, thalidomide, dexamethasone) regimen in patients with multiple myeloma--a report by the Polish Myeloma Study Group. Leuk Res.

[14] Morgan GJ, Davies FE, Gregory WM, Russell NH, Bell SE, Szubert AJ, Navarro Coy N, Cook G, Feyler S, Byrne JL, Roddie H, Rudin C, Drayson MT (2011). Cyclophosphamide, thalidomide, and dexamethasone (CTD) as initial therapy for patients with multiple myeloma unsuitable for autologous transplantation. Blood.

[15] Hungria VT, Crusoé EQ, Maiolino A, Bittencourt R, Fantl D, Maciel JF, Pessoa de Magalhaes RJ, Almeida MS, Cury P, Hisgashi F, Peres AL, Chiattone CS (2016). Phase 3 trial of three thalidomide-containing regimens in patients with newly diagnosed multiple myeloma not transplant-eligible. Ann Hematol.

[16] van de Velde HJ, Liu X, Chen G, Cakana A, Deraedt W, Bayssas M (2007). Complete response correlates with long-term survival and progression-free survival in high-dose therapy in multiple myeloma. Haematologica.

[17] Harousseau JL, Attal M, Avet-Loiseau H (2009). The role of complete response in multiple myeloma. Blood.

[18] Kastritis E, Zervas K, Symeonidis A, Terpos E, Delimbassi S, Anagnostopoulos N, Michali E, Zomas A, Katodritou E, Gika D, Pouli A, Christoulas D, Roussou M (2009). Improved survival of patients with multiple myeloma after the introduction of novel agents and the applicability of the International Staging System (ISS): an analysis of the Greek Myeloma Study Group (GMSG). Leukemia.

[19] Iriuchishima H, Saitoh T, Handa H, Isoda A, Matsumoto M, Sawamura M, Iwasaki A, Ushie C, Hattori H, Sasaki Y, Mitsui T, Yokohama A, Tsukamoto N (2015). A new staging system to predict prognosis of patients with multiple myeloma in an era of novel therapeutic agents. Eur J Haematol.

[20] Augustson BM, Begum G, Dunn JA, Barth NJ, Davies F, Morgan G, Behrens J, Smith A, Child JA, Drayson MT (2005). Early mortality after diagnosis of multiple myeloma: analysis of patients entered onto the United kingdom Medical Research Council trials between 1980 and 2002--Medical Research Council Adult Leukaemia 145 Working Party. J Clin Oncol.

[21] Eleutherakis-Papaiakovou V, Bamias A, Gika D, Simeonidis A, Pouli A, Anagnostopoulos A, Michali E, Economopoulos T, Zervas K, Dimopoulos MA, Greek Myeloma Study Group (2007). Renal failure in multiple myeloma: incidence, correlations, and prognostic significance. Leuk Lymphoma.

[22] Kumar SK, Rajkumar SV, Dispenzieri A, Lacy MQ, Hayman SR, Buadi FK, Zeldenrust SR, Dingli D, Russell SJ, Lust JA, Greipp PR, Kyle RA, Gertz MA (2008). Improved survival in multiple myeloma and the impact of novel therapies. Blood.

[23] Jung SH, Cho MS, Kim HK, Kim SJ, Kim K, Cheong JW, Kim SJ, Kim JS, Ahn JS, Kim YK, Yang DH, Kim HJ, Lee JJ (2016). Risk factors associated with early mortality in patients with multiple myeloma who were treated upfront with a novel agents containing regimen. BMC Cancer.

[24] Greipp PR, San Miguel J, Durie BG, Crowley JJ, Barlogie B, Bladé J, Boccadoro M, Child JA, Avet-Loiseau H, Kyle RA, Lahuerta JJ, Ludwig H, Morgan G (2005). International staging system for multiple myeloma. J Clin Oncol.

[25] Bringhen S, Mateos MV, Zweegman S, Larocca A, Falcone AP, Oriol A, Rossi D, Cavalli M, Wijermans P, Ria R, Offidani M, Lahuerta JJ, Liberati AM (2013). Age and organ damage correlate with poor survival in myeloma patients: meta-analysis of 1435 individual patient data from 4 randomized trials. Haematologica.

[26] Boyd KD, Ross FM, Chiecchio L, Dagrada G, Konn ZJ, Tapper WJ, Walker BA, Wardell CP, Gregory WM, Szubert AJ, Davies FE, Morgan GJ (2011). Gender disparities in the tumor genetics and clinical outcome of multiple myeloma. Cancer Epidemiol Biomarkers Prev.

[27] Chang WJ, Kang ES, Lee ST, Kim SH, Kim DW, Kim SJ, Kim K (2014). Thalidomide, cyclophosphamide and dexamethasone induction therapy: feasibility for myeloma patients destined for autologous stem cell transplantation. Acta Haematol.

[28] Beason TS, Chang SH, Sanfilippo KM, Luo S, Colditz GA, Vij R, Tomasson MH, Dipersio JF, Stockerl-Goldstein K, Ganti A, Wildes T, Carson KR (2013). Influence of body mass index on survival in veterans with multiple myeloma. Oncologist.

[29] Singhal S, Mehta J, Desikan R, Ayers D, Roberson P, Eddlemon P, Munshi N, Anaissie E, Wilson C, Dhodapkar M, Zeddis J, Barlogie B (1999). Antitumor activity of thalidomide in refractory multiple myeloma. N Engl J Med.

[30] Offidani M, Corvatta L, Polloni C, Piersantelli MN, Galieni P, Visani G, Alesiani F, Catarini M, Brunori M, Burattini M, Centurioni R, Ferranti M, Giuliodori L (2008). Serum C-reactive protein at diagnosis and response to therapy is the most powerful factor predicting outcome of multiple myeloma treated with thalidomide/anthracycline-based therapy. Clin Lymphoma Myeloma.

[31] Barlogie B, Desikan R, Eddlemon P, Spencer T, Zeldis J, Munshi N, Badros A, Zangari M, Anaissie E, Epstein J, Shaughnessy J, Ayers D, Spoon D (2001). Extended survival in advanced and refractory multiple myeloma after single-agent thalidomide: identification of prognostic factors in a phase 2 study of 169 patients. Blood.

[32] Ozkurt ZN, Yağci M, Sucak GT, Kirazli S, Haznedar R (2010). Thrombopoietic cytokines and platelet count in multiple myeloma. Platelets.

[33] Kamińska J, Koper OM, Mantur M, Matowicka-Karna J, Sawicka-Powierza J, Sokołowski J, Kostur A, Kulczyńska A, Kłoczko J, Kemona H (2014). Does thrombopoiesis in multiple myeloma patients depend on the stage of the disease?. Adv Med Sci.

[34] Huang SY, Lin CW, Lin HH, Yao M, Tang JL, Wu SJ, Chen YC, Lu HY, Hou HA, Chen CY, Chou WC, Tsay W, Chou SJ (2014). Expression of cereblon protein assessed by immunohistochemicalstaining in myeloma cells is associated with superior response of thalidomide- and lenalidomide-based treatment, but not bortezomib-based treatment, in patients with multiple myeloma. Ann Hematol.

[35] Jung SH, Choi HJ, Shin MG, Lee SS, Hwang EC, Jung TY, Cho MS, Yang DH, Ahn JS, Kim YK, Kim HJ, Lee JJ (2016). Thalidomide-based induction regimens are as effective as bortezomib-based regimens in elderly patients with multiple myeloma with cereblon expression. Ann Hematol.

[36] Iskierka-Jazdzewska E, Stepien A, Canzian F, Martino A, Campa D, Stein A, Krawczyk-Kulis M, Rybicka M, Kyrcz-Krzemien S, Butrym AK, Mazur G, Jurczyszyn AJ, Zawirska D (2014). Cereblon (CRBN) Gene polymorphisms predict clinical response and progression-free survival in multiple myeloma patients treated with lenalidomide: a pharmacogenetic study of immense consortium. Blood.

[37] Butrym A, Łacina P, Rybka J, Chaszczewska-Markowska M, Mazur G, Bogunia-Kubik K (2016). Cereblon and IRF4 variants affect risk and response to treatment in multiple myeloma. Arch Immunol Ther Exp (Warsz).

